# Use of an unmanned aircraft system to quantify NO_*x*_ emissions from a natural gas boiler

**DOI:** 10.5194/amt-14-975-2021

**Published:** 2021-02-09

**Authors:** Brian Gullett, Johanna Aurell, William Mitchell, Jennifer Richardson

**Affiliations:** 1 US Environmental Protection Agency, Office of Research and Development, Research Triangle Park, North Carolina 27711, USA; 2 University of Dayton Research Institute, Dayton, Ohio 45469-7532, USA; 3 The Dow Chemical Company, Midland, Michigan 48667, USA

## Abstract

Aerial emission sampling of four natural gas boiler stack plumes was conducted using an unmanned aerial system (UAS) equipped with a lightweight sensor–sampling system (the “Kolibri”) for measurement of nitrogen oxide (NO), and nitrogen dioxide (NO_2_), carbon dioxide (CO_2_), and carbon monoxide (CO). Flights (*n*=22) ranged from 11 to 24min in duration at two different sites. The UAS was maneuvered into the plumes with the aid of real-time CO_2_ telemetry to the ground operators and, at one location, a second UAS equipped with an infrared–visible camera. Concentrations were collected and recorded at 1Hz. The maximum CO_2_, CO, NO, and NO_2_ concentrations in the plume measured were 10000, 7, 27, and 1.5 ppm, respectively. Comparison of the NO_*x*_ emissions between the stack continuous emission monitoring systems and the UAS–Kolibri for three boiler sets showed an average of 5.6% and 3.5% relative difference for the run-weighted and carbon-weighted average emissions, respectively. To our knowledge, this is the first evidence of the accuracy performance of UAS-based emission factors against a source of known strength.

## 1 Introduction

Aerial measurement of plume concentrations is a new field made possible by advances in unmanned aircraft systems (UASs, or “drones”), miniature sensors, computers, and small batteries. The use of a UAS platform for environmental sampling has significant advantages in many scenarios in which access to environmental samples is limited by location or other factors. Hazards to equipment and personnel can also be minimized by the mobility of UASs and their ability to be remotely operated away from hazardous sources. UAS-based emission samplers have been used for measurement of area source gases (Neumann et al., 2013; Rosser et al., 2015; Chang et al., 2016; Li et al., 2018), point source gases (Villa et al., 2016), aerosols (Brady et al., 2016), black carbon particles (Craft et al., 2014), volcanic pollutants (Mori et al., 2016), particle mass (Peng et al., 2015), and particle number concentrations (Villa et al., 2016).

UAS-based emission measurements are particularly suited for area source measurements of fires and can be used to determine emission factors, or the mass amount of a pollutant per unit of source operation, such as the mass of particulate matter (PM) per mass of fuel (e.g., biomass) burned. These values can be converted into emission rates, such as the mass of pollutant per unit of energy (e.g., gNO_*x*_ kJ^−1^). These determinations typically rely on the carbon balance method in which the target pollutant is co-sampled with the major carbon species present, and, with knowledge of the source’s fuel (carbon) composition, the pollutant-to-fuel ratio or an emission rate and/or factor can be calculated.

For internal combustion sources that have a process emission stack, downwind plume sampling can use the same method. When combined with the source fuel supply rate and stack flow rates (to determine the dilution rate), measurements comparable to extractive stack sampling may be possible. To our knowledge, determination of emission factors from a stack plume using a UAS-borne sampling system has not been previously demonstrated. The goal of this effort was to compare NO_*x*_ measurements obtained by UAS-borne emission samplers to those from concurrent continuous emission monitoring (CEM) measurements. While not necessarily obviating the need for CEM for regulatory compliance, the use of UAS-based measurements could provide a safe and fast method of checking emissions that does not require personnel and equipment to access elevated stacks for periodic CEM verification. More importantly, however, the comparison of UAS-based emission measurements against a source of known CEM-determined concentration allows the accuracy of this new type of measurement to be assessed. Demonstrating the efficacy of these measurements would then open their applicability to other less understood sources that are not amenable to conventional CEM sampling, such as open fires, industrial flares, and gas releases.

The feasibility of downwind plume sampling using a sensor-equipped UAS was tested on industrial boilers at the Dow Chemical Company (Dow) facilities in Midland, Michigan (MI), and St. Charles, Louisiana (LA). The sensor system was designed and built by the EPA’s Office of Research and Development, and the UAS was owned and flown by the Dow Corporate Aviation Group. To determine the comparative accuracy of the measurements, the UAS-based emission factor was compared to the stack continuous emission monitoring systems (CEMSs). The target pollutants were nitrogen oxide (NO) and nitrogen dioxide (NO_2_) to mimic the stack CEMS measurement methods. Carbon as carbon dioxide (CO_2_) and carbon monoxide (CO) was measured on the UAS for the carbon balance method.

## 2 Materials and methods

Plume sampling tests were conducted on two natural-gas-fired industrial boilers located at Dow’s Midland, Michigan, and St. Charles, Louisiana, facilities. The Midland boilers are fire-tube-type boilers using low-pressure utility-supplied natural gas. They are equipped with low NO_*x*_ burners and utilize flue gas recirculation to reduce stack NO_*x*_ concentrations. The Midland facility burned natural gas with a higher heating value (HHV) of 9697kcalm^−3^ (1089 BTU ft^−3^: British Thermal Units per cubic foot). The two tested stacks are 14m above ground level and 7m apart. To avoid sampling overlapping plumes, only a single boiler was operating during the testing. The St. Charles boilers are D-type water package boilers using natural gas fuels (high-pressure fuel gas – HPFG; low-pressure off-gas – LPOG). They are equipped with low NO_*x*_ burners with flue gas recirculation to reduce stack NO_*x*_ concentrations. The boiler stacks are about 20m apart and reach over 20m in height above ground level. The St. Charles facility burned natural gas under steady-state conditions with a composition of 77.12% CH_4_, 2.01% C_2_H_6_, and 19.91% H_2_, with an HHV of 7845 kcal m^−3^ (881 BTU ft^−3^). Both boilers were operational during aerial sampling, but the wind direction and UAS proximity to the target stack precluded co-mingling of the plumes.

Air sampling was accomplished with an EPA–ORD-developed sensor–sampler system termed the “Kolibri”. The Kolibri consists of real-time gas sensors and pump samplers to characterize a broad range of gaseous and particle pollutants. This self-powered system has a transceiver for data transmission and pump control (Xbee S3B, Digi International, Inc., Minnetonka, MN, USA) from the ground-based operator. For this application, gas concentrations were measured using electrochemical cells for CO, NO, and NO_2_ and a non-dispersive infrared (NDIR) cell for CO_2_ (Table 1). All sensors were selected for their applicability to the anticipated operating conditions of concentration level and temperature as well as for their ability to rapidly respond to changing plume concentrations due to turbulence and entrainment of ambient air. Each sensor underwent extensive laboratory testing to verify performance and suitability prior to selection for the Kolibri. Tests included sensor performance (linearity, drift, response time, noise, detection limits) in response to anticipated field temperatures, pressure, humidity, and interferences. Additional information from the manufacturers on sensor performance is available from the links in Table 1. In anticipation of temperatures as low as 0 °C at the Midland site and to avoid daily temperature fluctuations, insulation was added to the Kolibri frame, and the sampled gases were preheated prior to the sensor with the use of a heating element and micro-fan inside the Kolibri. All sensors were calibrated before each sampling day under local ambient conditions. After sampling was completed, the sensors were similarly tested to assess potential drift.

Concentration data were stored by the Kolibri using a Teensy USB-based microcontroller board (Teensy 3.2, PJRC, LLC, Sherwood, OR, USA) with an Arduino-generated data program and secure digital data card. All four sensors underwent pre- and post-sampling two- or three-point calibration using gases (Calgasdirect Inc., Huntington Beach, CA, USA) traceable to National Institute of Standards and Technology (NIST) standards.

The NO sensor (NO-D4) is an electrochemical gas sensor (Alphasense, Essex, UK) that measures concentration by changes in impedance. The sensor has a detection range of 0 to 100 ppm with a resolution of < 0.1 root mean square (rms) noise (parts per million equivalent) and linearity error within ± 1.5 ppm at full scale. The NO-D4 was tested to have a response time to reach 95% of the reference concentration (*t*_95_) of 6.3 ± 0.52 s and a noise level of 0.027 ppm. The temperature and relative humidity (RH) operating range is 0 to +50 °C and 15% to 90% RH, respectively.

The NO_2_ sensor (NO2-D4) is an electrochemical gas sensor (Alphasense, Essex, UK) that likewise measures by impedance changes. It has an NO_2_ detection range of 0–10 ppm with a resolution of 0.1 rms noise (parts per million equivalent) and linearity error of 0 to 0.6 ppm at full scale. Its *t*_95_ was measured as 32.3 ± 3.8s with a noise level of 0.015 ppm. The temperature and RH operating range is 0 to +50 °C and 15% to 90% RH, respectively.

Laboratory calibration testing prior to field measurements on both the NO-D4 and NO2-D4 sensor outputs showed their responses to be linearly proportional (*R*^2^ > 0.99) over the range of four- and five-point calibration gas concentrations. The response times of both sensors were derived using the maximum reference concentration of 47.81 ppm for NO and 10.46 ppm of NO_2_. The times to reach 95% of the reference concentration, *t*_95_, were 6.3 and 32.3s (relative standard deviation: RSD 8.2% and 11.8%), respectively, for the NO-D4 and NO2-D4 sensors. These response times are both shorter than those measured simultaneously in the laboratory with CEM (Ametek 9000 RM, Pittsburgh, PA, USA) at 37 and 50s, respectively, for NO and NO_2_.

The CO_2_ sensor (CO_2_ Engine® K30 Fast Response, SenseAir, Delsbo, Sweden) is an NDIR gas sensor, and the voltage output is linear from 400 to 10000 ppm. The temperature and RH operating range is 0 to +50 °C and 0% to 90% RH, respectively. The CO_2_-K30 sensor was measured to have a *t*_95_ response time at 6000 ppm CO_2_ of 9.0 ± 0.0s and a noise level of 1.6 ppm. The response time was 4s longer than compared to CO_2_ measured by a portable gas analyzer (LI-820, LI-COR Biosciences, Lincoln, NE, USA). The sensor and the LI-820 showed good agreement, as the measurements showed an *R*^2^ of 0.99 and a slope of 1.01.

The CO sensor (e2V EC4-500-CO, SGX Sensortech Ltd, High Wycombe, Buckinghamshire, UK) is described more fully elsewhere (Aurell et al., 2017; Zhou et al., 2017). In previous sensor evaluation tests with laboratory biomass burns (Zhou et al., 2017) with CO ranging between 0 and 250 ppm, the sensor was compared to simultaneous measurements by a CO continuous emission monitor (CAI model 200, California Analytical Instruments Inc., Orange, CA, USA). The concentration measurements had an *R*^2^=0.98 and a slope of 1.04, indicating the level of agreement between the two devices. The *t*_90_ was measured as 18s, while the time-integrated CO concentration differences with the CAI-200, rated at *t*_90_ < 1s, were only 4.9%.

Variations of the Kolibri sampling system allow for measurement of additional target pollutants. These include particulate matter (PM), polycyclic aromatic hydrocarbons (PAHs), and volatile organic compounds (VOCs), including carbonyls, energetics, chlorinated organics, metals from filter analyses, and perchlorate (Aurell et al., 2017; Zhou et al., 2017).

**Figure 1.**
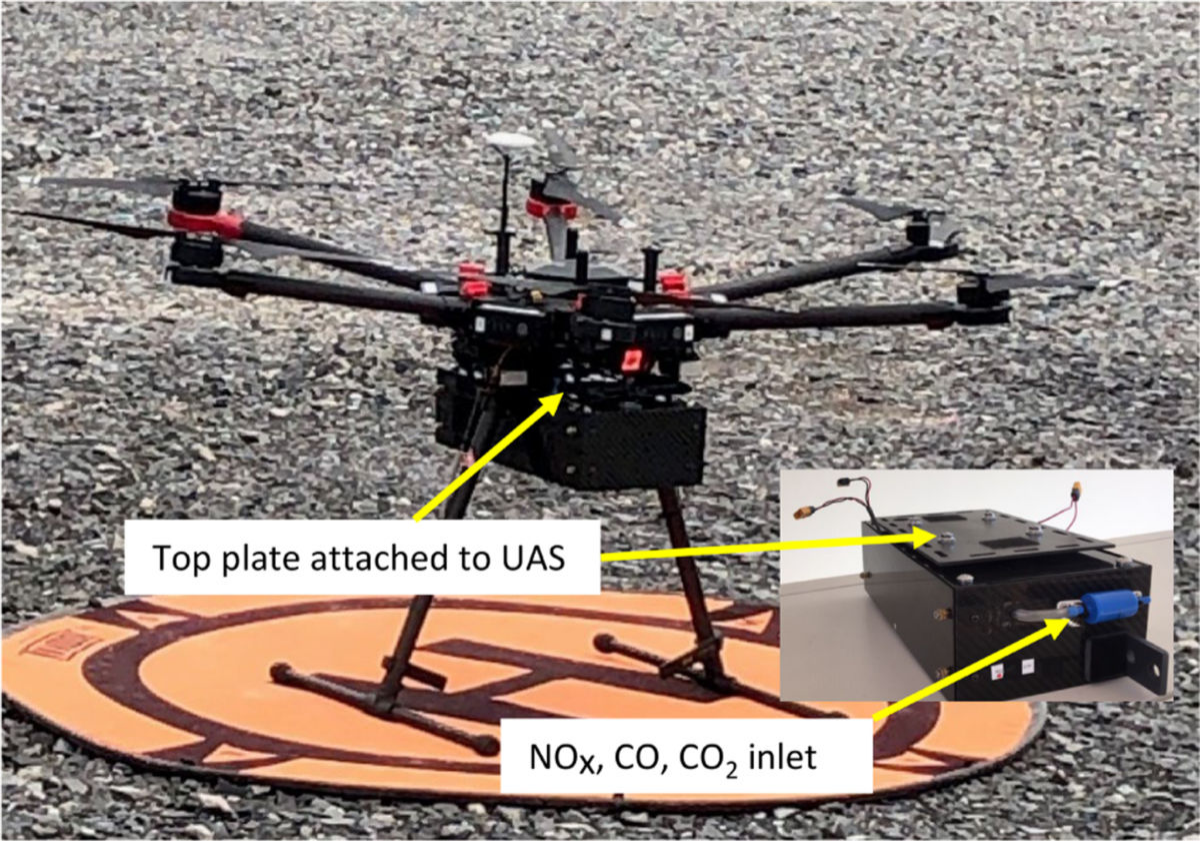
Dow UAS with Kolibri attached to the undercarriage.

At both facilities the aviation team from Dow flew their DJI Matrice 600 UAS, a six-motor multicopter (hexacopter), into the plumes with an EPA–ORD Kolibri sensor–sampler system attached to the undercarriage (Fig. 1). In this configuration of sensors, the Kolibri system weighed 2.4kg. Typical flight elevations at Midland and St. Charles were 21 and 32m above ground level (a.g.l.), respectively, and flight durations ranged from 9 to 24min. At the St. Charles location, the UAS pilot was approximately 100m from the center point of the two stacks, easily allowing for line-of-sight operation. A telemetry system on the Kolibri provided real-time CO_2_ concentration and temperature data to the Kolibri operator, who in turn advised the pilot on the optimum UAS location.

CEMSs on the boiler stacks produced a continuous record of NO_*x*_ emissions and O_2_ concentrations. Stack and CEMS types located at the Midland and St. Charles facilities are shown in Table 2. The stack NO_*x*_ analyzer uses a chemiluminescence measurement with a photomultiplier tube and is capable of split concentration range operation: low (0–180 ppm) and high (0–500 ppm). Its response time is reported as 5s. The O_2_ analyzer uses a zirconium oxide cell with a measurement range of 0% to 25% and a reported *t*_95_ of < 10s.

The plant CEMSs undergo annual relative accuracy audit testing (NSPS Subpart Db, Part 70) using US EPA Method 7E (2014) for NO_*x*_ and US EPA Method 3A (2017) for O_2_. Calculation of NO_*x*_ emissions uses the appropriate F factor, a value that relates the required combustion gas volume to fuel energy input, as described in US EPA Method 19 (2017). Flue gas analysis for O_2_ and CO_2_ is performed in accordance with US EPA Method 3A (2017) using an infrared analyzer to allow for calculation of the flue gas dry molecular weight.

The CEMS and UAS–Kolibri data were reduced to a common basis for comparison of results. Emission factors, or the mass of NO_*x*_ per mass of fuel carbon burned, and emission rates, or the mass of NO_*x*_ per energy content of the fuel, were calculated from the sample results. The determination of emission factors, defined as the mass of pollutant per mass of fuel burned, depends upon foreknowledge of the fuel composition, specifically its carbon concentration and its supply rate. The carbon in the fuel is presumed for calculation purposes to proceed to either CO_2_ or CO, with the minor carbon mass in hydrocarbons and PM ignored for this source type. Concurrent emission measurements of pollutant mass and carbon mass (as CO_2_+CO) can be used to calculate total emissions of the pollutant from the fuel using its carbon concentration and fuel burn rate.

The UAS–Kolibri emission factors were calculated from the mass ratio of NO+NO_2_, with the mass of CO+CO_2_ resulting in a value with units of mgNO_*x*_ kg^−1^ C. CO_2_ concentrations were corrected for upwind background concentrations. CEMS values of O_2_ and fuel flow rate were used to calculate stack flow rate using US EPA Method 19 (2017). This method requires the fuel higher heating value and an F factor (gas volume per fuel energy content, e.g., m^3^ kcal^−1^, ft^3^ BTU^−1^) to complete the calculation. For natural gas, the F factor is 967m^3^ 10^−6^ kcal (8710 ft^3^ 10^−6^ BTU) (Table 19–2, US EPA Method 19, 2017). The concentration, stack flow rate, and fuel flow rate data allow for the determination of NO_*x*_ and C emission rates.

## 3 Results and discussion

**Figure 2.**
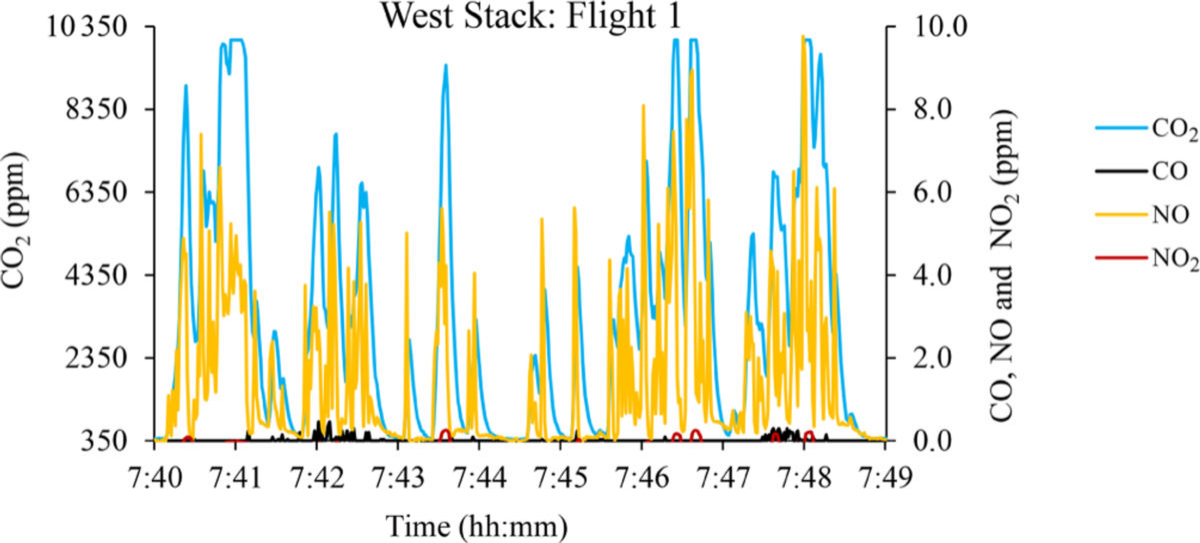
Example of UAS–Kolibri-measured plume concentrations from the St. Charles west boiler. Data reported at 1Hz.

The UAS–Kolibri team easily found the stack plumes at both locations using the wind direction and CO_2_ telemetry data transmitted to the ground operator. Use of an infrared–visible (IR: infrared) camera on a second UAS at St. Charles for some of the flights aided more rapid location of the plume and positioning of the UAS–Kolibri. Gas concentration fluctuations were rapid and of high magnitude as observed in a representative trace in Fig. 2. CO_2_ concentrations up to 10000 ppm were observed; the relatively lower average CO_2_ concentrations reflect the rapid mixing and entrainment of ambient air, causing dilution.

Sampling data and emission factors from the UAS–Kolibri are shown in Tables 3, 4, and 5 for the Midland, St. Charles east stack, and St. Charles west stack, respectively. Eight sampling flights were conducted at the Midland site; five were conducted on the St. Charles east boiler and nine on the St. Charles west boiler. Both boilers at the Midland site were operated under the same conditions, so their results have been presented together. Flight times averaged 14min (10% RSD) at the Midland facility and just over 20min (10% RSD) at the St. Charles facility. The shorter flight times in Midland were due to lower UAS battery capacity caused by colder temperatures (the sampling temperatures in the plume averaged 10 ± 3 °C). The average multi-concentration drift for each of the sensors, tested at both locations after each sampling day, was less than ± 3%. The NO2-D4 sensor showed higher drift (average 8.6%) at one location for the highest concentration of its calibration gas (10.4 ppm). This had a minimal effect on the emission factor calibrations as the measured NO_2_ in the plume was actually less than 1 ppm, a range in which the drift was much lower, and NO_2_ is a minor contributor to the measured NO_*x*_ species.

Average plume NO_*x*_ concentrations were 0.88 ± 0.32 ppm at Midland and 1.22 ppm and 2.41 ppm at the two St. Charles boilers, with an average RSD of 37%, 36%, and 12%, respectively. The NO emission factor was typically 97% of the total NO_*x*_, with the NO_2_ providing the minor balance.

Table 6 presents the average O_2_ and NO_*x*_ measurement results and the fuel supply rate at both locations. Values for natural gas supply, adjusted for the C_2_H_6_ and H_2_ composition of the St. Charles fuel, were used to calculate the fuel carbon supply rate. These data allow for the calculation of the emission factor, or the mass of NO_*x*_ to the mass of carbon, which is reported in Table 7.

The UAS–Kolibri NO_*x*_ emission factor for Midland is 8% higher than the simultaneous CEMS value. For the east and west boilers at St. Charles, the UAS–Kolibri NO_*x*_ emission factor value is < 1% and 8% higher, respectively, than the CEMS values. The difference for the UAS–Kolibri in Midland may be attributed in part to the extremely cold temperature affecting the performance of the electrochemical sensors. The standard deviations for the CEMS data are based on the run-averaged NO_*x*_ values for each test. These values were calculated based on 10s averaging for the Midland tests, 60s averaging in St. Charles, and 1s averaging for the UAS–Kolibri. Higher standard deviations for the UAS–Kolibri are predictable given the rapidly changing values and wide range (∼0–10 ppm) of NO_*x*_ data observed in Fig. 2. Difference testing for the CEMS and UAS–Kolibri using *α* =0.05 and assumed unequal variances indicates that only the west boiler and UAS–Kolibri are statistically distinct.

The emission rates calculated from the UAS–Kolibri data are 5.6, 14.6, and 13.3 kg NO_*x*_ × 10^−3^ kJ (0.013, 0.034, and 0.031 lb NO_*x*_ × 10^−6^ BTU), respectively, for the Midland, east St. Charles, and west St. Charles boilers, which are below the regulatory standard of 15.5 kg NO_*x*_ × 10^−3^ kJ (0.036 lb NO_*x*_ × 10^−6^ BTU). The emission factors were also calculated as carbon-weighted values to reflect potential differences in plume sampling efficiency between runs. The Midland, east St. Charles, and west St. Charles UAS–Kolibri emission factors were 607, 1525, and 1409 mg NO_*x*_ kg^−1^ C, respectively. These amounted to relative percent differences of 0.8%, 1.9%, and 7.8% between the CEM and UAS–Kolibri values for an overall run-weighted average difference of 5.6%. The difference between the CEM readings and those from the Kolibri weighted by the carbon collection amounts, reflecting success at being within the higher plume concentrations, was 3.5%.

## 4 Conclusions

This work reports, to our knowledge, the first known comparison of continuous emission monitoring measurements made in a stack to downwind plume measurements made using a UAS equipped with emission sensors.

The UAS–Kolibri system was easily able to find and take measurements from the downwind plume of a natural gas boiler despite the lack of any visible plume signature. The telemetry system aboard the Kolibri reported real-time CO_2_ concentrations to the operator on the ground, allowing the operator to provide immediate feedback to the UAS pilot on plume location. Comparison of the CEM data to the UAS–Kolibri data from field measurements at two locations showed agreement of NO_*x*_ emission factors within 5.6% and 3.5% for time-weighted and carbon-collection-weighted measurements, respectively. This work demonstrates the accuracy of a UAS-borne emission sampling system for quantifying point source strength. These results also have applicability to area source measurements, such as open fires, which similarly employ the carbon balance method to determine source strength emission factors.

## Figures and Tables

**Table 1.** UAS–Kolibri target analytes and methods.

Analyte	Instrument, manufacturer data link (last access: 25 January 2021)	Frequency	Cal. gases (ppm) Midland	Cal. gases (ppm) St. Charles
CO_2_	SenseAir CO_2_ Engine K30, NDIR^a^ https://www.co2meter.com/products/k-30-co2-sensor-module	Continuous, 1 Hz^b^	408, 990	392, 996, 5890
CO	E2v EC4-500-CO, electrochemical cell https://www.sgxsensortech.com/content/uploads/2014/07/EC4-500-CO1.pdf	Continuous, 1Hz	0^c^, 9.67, 50.6	0, 9.9, 51.8
NO	NO-D4, electrochemical cell http://www.alphasense.com/WEB1213/wp-content/uploads/2020/12/NO-D4.pdf	Continuous, 1Hz	0, 2.1, 41.4	0, 2.1, 40.4
NO_2_	NO2-D4, electrochemical cell http://www.alphasense.com/WEB1213/wp-content/uploads/2020/12/NO2-D4.pdf	Continuous, 1Hz	0, 2.1, 10.4	0, 1.9, 10.4

a Non-dispersive infrared.

b Hz –hertz.

c Zero (0) cal. gas: air.

**Table 2.** CEMS instruments at both Dow locations.

Gas measured	Midland CEMS	St. Charles CEMS
O_2_	Gaus model 4705	ABB/Magnos 106
NO*x*	Thermo model 42i-HL	ABB/Limas 11

**Table 3.** Midland UAS–Kolibri sampling data and emission factors. Time is indicated in US Central Standard Time (GMT−6).

Date (mm/dd/yyyy)	Flight No.	Flight time (hh:mm:ss)			NO_2_ mg kg^−1^ C	NO mg kg^−1^ C	NO_*x*_ mg kg^−1^ C	Avg. CO_2_ ppm
		Up	Down	Total				
11/14/2018	1	10:29:00	10:43:00	00:14:00	201	618	819	1213
11/14/2018	2	11:13:04	11:28:28	00:15:24	186	624	810	1138
11/14/2018	3	12:54:17	13:08:47	00:14:30	230	659	889	2948
11/14/2018	5	13:27:40	13:42:05	00:14:25	99	570	669	4658
11/15/2018	6	10:24:20	10:39:30	00:15:10	61	394	454	3703
11/15/2018	7	10:41:36	10:52:40	00:11:04	84	397	481	3983
11/15/2018	8	10:55:10	11:10:10	00:15:00	126	398	524	4781

Average				00:14:13	141	523	664	3203
SD				00:01:28	65	121	179	1514
RSD (%)				10	46	23	27	47

Flight no. 4 was excluded from calculations as CO was observed, which originated from a cycling second boiler.

**Table 4.** St. Charles east stack UAS–Kolibri sampling data and emission factors. Time is indicated in US Central Daylight Time (GMT–5).

Date (mm/dd/yyyy)	Flight No.	Flight time (hh:mm:ss)			NO_2_ mg kg^−1^ C	NO mg kg^−1^ C	NO_*x*_ mg kg^−1^ C	Avg. CO_2_ ppm
		Up	Down	Total				
07/23/2019	1	09:49:00	10:07:00	00:18:00	1	1442	1442	2305
07/23/2019	2	10:12:00	10:34:00	00:22:00	15	1461	1476	2526
07/23/2019	3	10:45:00	11:08:00	00:23:00	5	1534	1539	785
07/23/2019	4	11:11:00	11:31:00	00:20:00	101	1684	1785	1082
07/23/2019	5	11:52:00	12:01:00	00:09:00	107	2110	2217	1923

Average				00:20:45	30	1530	1560	1675
SD				00:02:13	47	110	155	869
RSD (%)				11	155	7.2	9.9	52

Flight no. 5 was not included in the average as elevated CO concentrations were detected, likely from other sources in the facility.

**Table 5.** St. Charles west stack UAS–Kolibri sampling data and emission factors. Time is indicated in US Central Daylight Time (GMT–5).

Date (mm/dd/yyyy)	Flight No.	Flight time (hh:mm:ss)			NO_2_ mg kg^−1^ C	NO mg kg^−1^ C	NO_*x*_ mg kg^−1^ C	Avg. CO_2_ ppm
		Up	Down	Total				
07/24/2019	1	07:31:00	07:49:00	00:18:00	25	1366	1391	3221
07/24/2019	2	07:52:00	08:16:00	00:24:00	49	1263	1312	3503
07/24/2019	3	08:19:00	08:38:00	00:19:00	87	1420	1507	3415
07/24/2019	4	09:23:00	09:46:00	00:23:00	65	1341	1406	4509
07/24/2019	5	09:49:00	10:11:00	00:22:00	47	1296	1343	4813
07/24/2019	6	10:16:00	10:36:00	00:20:00	52	1299	1351	3773
07/24/2019	7	10:38:00	11:00:00	00:22:00	53	1316	1369	4194
07/24/2019	8	11:51:00	12:13:00	00:22:00	90	1460	1549	3129
07/24/2019	9	13:17:00	13:39:00	00:22:00	47	1464	1511	3606

Average				00:21:20	57	1358	1416	3796
SD				00:01:56	21	74	86	586
RSD (%)				9	36	5.5	6.0	15

**Table 6.** Multi-run average stack CEMS data.

	Midland Both boilers	St. Charles	
		East boiler	West boiler
O_2_ (%)	8.2	4.9	4.5
NO_*x*_ (ppm)	15.7	50.4	42.9
Fuel rate	39.3 × 10^6^ kJ h^−1^	155.2 × 10^6^ kJ h^−1^	177.8 × 10^6^ kJ h^−1^

**Table 7.** Comparison of average NO_*x*_ emission factors from CEMS and UAS–Kolibri.

Run-averaged NO_*x*_ emission factor (mg NO_*x*_ kg^−1^ C; ± 1 SD)			

	Midland Both boilers	St. Charles	
		East boiler	West boiler
CEMS	612 ± 10	1555 ± 50	1303 ± 29
UAS–Kolibri	664 ± 179	1560 ± 155	1416 ± 86
RPD: CEM & UAS–Kolibri, %	8.2	0.3	8.3
